# First Step Towards Larger DNA-Based Assemblies of Fluorescent Silver Nanoclusters: Template Design and Detailed Characterization of Optical Properties

**DOI:** 10.3390/nano9040613

**Published:** 2019-04-13

**Authors:** Liam E. Yourston, Alexander Y. Lushnikov, Oleg A. Shevchenko, Kirill A. Afonin, Alexey V. Krasnoslobodtsev

**Affiliations:** 1 Department of Physics, University of Nebraska Omaha, Omaha, NE 68182, USA; lyourston@unomaha.edu; 2 Nanoimaging Core Facility at the University of Nebraska Medical Center, Omaha, NE 68198, USA; alushnikov@unmc.edu; 3 Nanoscale Science Program, Department of Chemistry, University of North Carolina at Charlotte, Charlotte, NC 28223, USA; oshevche@uncc.edu (O.A.S.); kafonin@uncc.edu (K.A.A.)

**Keywords:** silver nanoclusters, fluorescence, i-motif DNA, cytosine rich sequences

## Abstract

Besides being a passive carrier of genetic information, DNA can also serve as an architecture template for the synthesis of novel fluorescent nanomaterials that are arranged in a highly organized network of functional entities such as fluorescent silver nanoclusters (AgNCs). Only a few atoms in size, the properties of AgNCs can be tuned using a variety of templating DNA sequences, overhangs, and neighboring duplex regions. In this study, we explore the properties of AgNCs manufactured on a short DNA sequence—an individual element designed for a construction of a larger DNA-based functional assembly. The effects of close proximity of the double-stranded DNA, the directionality of templating single-stranded sequence, and conformational heterogeneity of the template are presented. We observe differences between designs containing the same AgNC templating sequence—twelve consecutive cytosines, (dC)_12_. AgNCs synthesized on a single “basic” templating element, (dC)_12_, emit in “red”. The addition of double-stranded DNA core, required for the larger assemblies, changes optical properties of the silver nanoclusters by adding a new population of clusters emitting in “green”. A new population of “blue” emitting clusters forms only when ssDNA templating sequence is placed on the 5′ end of the double-stranded core. We also compare properties of silver nanoclusters, which were incorporated into a dimeric structure—a first step towards a larger assembly.

## 1. Introduction

The field of nucleic acid (NA) nanotechnology has brought various designs of materials and devices created with facilitation of nucleic acids both DNA and RNA [1,2,3,4]. The progress already made in the field makes nucleic acid molecules very promising candidates for fabrication of complex shapes and functional structures at the nanoscale. The NA nanotechnology toolbox utilizes not only traditional DNA and RNA bases but also their chemical analogs and backbone modifications, for example peptide nucleic acid (PNA) [5]. Additional flexibility is added by the architectural elements of NA that employ tertiary interactions, such as kissing loops [6,7], loop receptor interactions [8], paranemic motifs [9], G-quadruplexes [10], and i-motifs [11], just to name a few. All these combined properties of NA provide a remarkable control over the versatile molecular structures that they can be programmed to assemble. NA nanotechnology opens large prospects for practical applications of novel materials design, diagnostics, and nanomedicine.

Besides being used as architectural elements for construction of complex nanostructures, NA can be utilized as scaffolds for hosting various functionalities including moieties with unique optical, electronic, and magnetic properties [12]. One example is the use of DNA template to arrange gold nanoparticles in a plasmonic linear array [12]. Silver nanoclusters (AgNCs) represent a family of such novel functionalities. AgNCs cannot exist on their own—they have to be stabilized with capping agents that (1) limit their size, (2) stabilize nanoclusters, and (3) modify their electronic properties. DNA oligonucleotides have been widely utilized for making AgNCs (reviewed in [13]). DNA protects the metallic core of the clusters and also determines the size and geometry of the AgNCs, which in turn dictates the optical properties and stability of the nanoclusters. The resultant silver nanoclusters are only a few atoms in size. Unique optical properties of AgNCs originate from their size. In this “few atoms” size regime, the continuous density of states breaks up into discrete energy levels in the nanoclusters. With discrete states, the nanoclusters resemble molecular-like behavior with strong fluorescence observed in the visible-near IR range of the spectrum [14]. Additionally, AgNCs are more resistant to photobleaching than widely used organic dyes, quantum dots, or fluorescent proteins, which makes these structures suitable for a plethora of practical applications, including optoelectronics and nanophotonics [15]. It is especially exciting because AgNCs can be naturally integrated with NA assemblies [16] and NA assemblies can be tracked with AgNCs formation [17]. Importantly, NA template sequence can be varied to produce fluorescent silver clusters of distinct optical properties [14].

While promising, AgNCs exhibit complex optical behavior including various fluorescence peaks, effects of templating and non-templating NA sequences [18,19,20,21]. Interactions between silver and NA bases might produce long-lived dark electronic states that can be used to enhance fluorescence detection [22]. The complexity of AgNC optical properties is also manifested by the possibility of direct and indirect excitation: Directly in the visible spectral range or indirectly via UV-excitation of DNA bases [23]. The structure and nature of optically active electronic states are poorly understood [24,25]. Therefore, a detailed characterization of these effects is needed to establish how AgNC function in larger assemblies. Recent efforts indicate that large scale photonic applications might benefit from arranging AgNC in DNA-templated arrays [26] and spatial control of nanocluster positioning [27].

In this article, we report a detailed characterization of C_12_-based templates designed for creation of larger NA assemblies. The design requires the presence of a double-stranded DNA region in close proximity to templating single-stranded sequences. Such proximity of dsDNA has a significant effect on optical properties of silver nanoclusters. We also observe the effect of directionality as optical properties of AgNCs depend strongly on whether C_12_-templating sequence is positioned on the 3′ or 5′ end of the double-stranded core. Multiple contiguous cytosines in the C_12_ templating sequence trigger the formation of non-canonical structures in the presence of silver atoms, and this modulates the fluorescence of AgNC to a large extent. Atomic Force Microscopy imaging provides an unambiguous proof of conformational heterogeneity of DNA templates. We correlate such conformational differences with optical heterogeneity of AgNCs. A detailed comparison of emissive properties of AgNCs between UV excitation and visible excitation also points to the complex heterogeneous nature of generated AgNCs. The designed constructs presented here can be further programmed into NA nanostructures and networks bearing multiple functionalities. We demonstrate a simple assembly of two nanoclusters into a linear construct, which is the first step towards larger patterned NA-based functional assemblies. Practical applications of such networks are discussed.

## 2. Materials and Methods

### 2.1. Materials

All DNA oligonucleotides were purchased from Integrated DNA Technologies, Inc. (Coralville, IA, USA) as desalted products and used without further purification. Oligonucleotide sequences are: (dC)_12_—CCCCCCCCCCCC;

ssSD9-OEC_12_—GAGATGCTAACATGGCTCTAGTCGACGATCCCCCCCCCCCC; ssSD9-BEC_12_—CCCCCCCCCCCCTGAGATGCTAACATGGCTCTAGTCGACGATCCCCCCCCCCCC; ssSD9-5′-OEC_12_: CCCCCCCCCCCCTGAGATGCTAACATGGCTCTAGTCGACGA; ssSD9-DimerC–TCAACATCAGTCTGATAAGCTATTTCGTCGACTAGAGCCATGTTAGCATCTC.

ssSD9-DimerC is complimentary to ssSD9-OEC_12_ and to the following two sequences making “forward” or “reverse” assembly:

ssSD9-DimerC-R-forward—TAGCTTATCAGACTGATGTTGATCCCCCCCCCCCC; ssSD9-DimerC-R-reverse CCCCCCCCCCCCTTAGCTTATCAGACTGATGTTGA.

Sodium borohydride was purchased from TCI (TCI America, Inc., Portland, OR, USA), all other reagents were purchased from Sigma-Aldrich (Sigma-Aldrich, Inc., St. Louis, MO, USA).

### 2.2. Synthesis of Ag-DNA Nanoclusters

In a typical preparation, DNA and AgNO_3_ aqueous solutions were mixed at 55 °C and incubated for 25 min at room temperature in the ammonium acetate buffer. Next, NaBH_4_ aqueous solution was added and samples were placed on ice and stirred vigorously. The final concentrations of the components were C_DNA_ = 10 μM, C_AgNO3_ = 100 μM, C_NaBH4_ = 100 μM, and C_NH4Ac_ = 50 mM. The solution then was incubated in the dark for 24 h at 4 °C.

For manufacturing the assembly, the duplet sequence was utilized. To avoid interference of the neighboring C_12_–C_12_ sequences, nanoclusters were first created on ssSD9-C_12_ or ssR21-C_12_ strands as described above and subsequently annealed onto the larger complimentary ssDNA. For annealing, the mix was subjected to 1 h thermal treatment in water bath at 40 °C, followed by cooling in an ice-water bath. For fluorescence and atomic force microscopy (AFM) measurements, DNA-AgNC samples were used without further purification.

### 2.3. Atomic Force Microscopy Imaging

AFM imaging of DNA-templated AgNCs was performed on MultiMode AFM Nanoscope IV system (Bruker Instruments, Santa Barbara, CA, USA) in tapping mode. Briefly, 5 μL of the DNA-templated AgNC solution were deposited on amino-propyl-silatrane (APS) modified mica [28] for a total of 2 min [29]. Excess sample was washed with DI water and gently dried under a flow of high purity argon gas and under vacuum overnight. AFM images in air were then recorded with a 1.5 Hz scanning rate using TESPA-300 probes (Bruker AFM Probes, Santa Barbara, CA, USA) The probes have ~320 kHz resonance frequency and a spring constant of about 40 N/m. Images were processed using the “FemtoScan Online” software package (Advanced Technologies Center, Moscow, Russia). Heights of the structures were measured using the “cross section” tool within the “FemtoScan Online” program. Height was measured as the highest point in the cross-section profile with the background subtracted.

### 2.4. Fluorescence Measurements

The excitation and emission spectra were acquired on a Cary Eclipse Fluorescence Spectrophotometer (Agilent Technologies, Santa Clara, CA, USA). In all the measurements, the concentration of DNA was kept the same at 10 µM. Experiments were carried out at a room temperature of ~22 °C. Fluorescence measurements were carried out in Sub-Micro Fluorometer Cell, model 16.40F-Q-10 (StarnaCells, Inc., Atascadero, CA, USA). Only freshly prepared solutions were used for spectroscopic measurements. The excitation–emission matrix spectra (EEMS) were recorded with 2 nm resolution. Fluorescence spectra were recorded with the emission wavelength range from 270 nm to 800 nm, and the initial excitation wavelength was set to 220 nm and final excitation wavelength was set to 700 nm with an increment of 2 nm. The slits were open to 5 µm and the PMT voltage was set to 700 V. EEMS data were then used for the 2D contour plot using MagicPlot Pro software (Magicplot Systems, LLC, Saint Petersburg, Russia).

## 3. Results

### 3.1. Design of AgNC DNA Template

**Figure 1:**
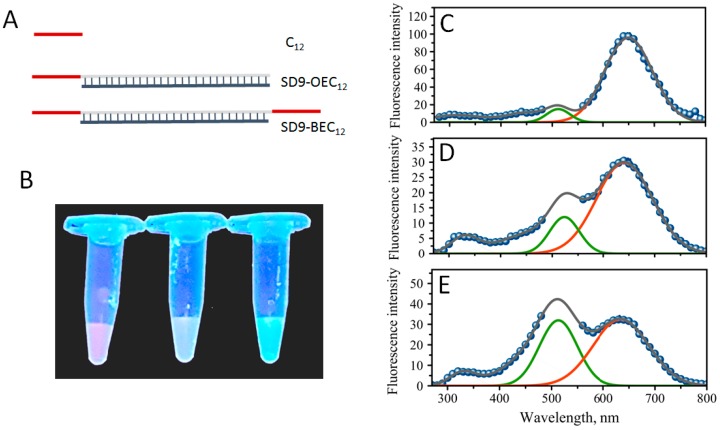
(**A**) Schematic representation of the template design, (**B**) a photograph of fluorescent glowing of solutions containing fluorescent silver nanoclusters (AgNC) templated on C_12_ (red), SD9-OEC_12_ (yellow), and SD9-BEC_12_ (green), (left to right) under the UV excitation on a trans-illuminator. Fluorescence spectra of AgNCs recorded with 254 nm excitation wavelength for the following templates (**C**) C_12_, (**D**) SD9-OEC_12_, and (**E**) SD9-BEC_12_. Gaps in the spectra are due to the removal of the second order scattering. Green and red solid lines are plotted as “guide for the eye” in the positions of major “green” and “red” emission peaks.

A number of DNA sequences that template and stabilize AgNCs have been studied (reviewed in [13]). Recently, it was proposed that identification of specific genome sequences can be made based on the properties of AgNCs that these sequences template [21]. The most common ones used in AgNC synthesis are cytosine-rich sequences due to the very high affinity of cytosines to silver cations [20,30]. Our primary goal was to characterize the properties of DNA-templated AgNC and explore the possibility of incorporating AgNCs into larger NA networks with functional properties via a bottom-up assembly. We have designed sequences consisting of two parts: one that templates AgNCs formation and another that is used for the further construction of the assembly. The designed sequences have a double-stranded core, schematically illustrated in Figure 1A, with a random 30 bp sequence, which does not form any secondary stable folds or hairpins [31]. AgNC templating sequence has a stretch of twelve consecutive cytosines—(dC)_12_, Figure 1A. This DNA construct can be used simultaneously as a template for AgNC formation and as a building block for making larger assemblies. Two sequences were initially studied: SD9 core modified with (dC)_12_ at only one end and SD9 core modified with (dC)_12_ at both ends. These sequences were named SD9-OEC_12_ and SD9-BEC_12_, respectively. Comparison with (dC)_12_ sequence alone–named C_12_ later in the text–was performed to elucidate the effect of the neighboring duplex region on the properties of AgNCs created with the single-stranded DNA template.

### 3.2. The Formation of Silver Nanoclusters and Their Optical Properties under UV Excitation

The formation of DNA-templated AgNCs is manifested by the changes in solution that are observed after 24 h incubation after the addition of Ag^+^ and sodium borohydride. Fluorescent glowing of the solutions is evident with UV excitation under a trans-illuminator. Figure 1B shows such glowing effect of AgNC-DNA solutions for three samples corresponding to sequences C_12_, SD9-OEC_12_, and SD9-BEC_12_. Since the templating sequence is the same for all three samples—C_12_ stretch, we expected similar colors. It appears, however, that all three samples have distinct fluorescence colors: Red for C_12_, yellow for SD9-OEC_12_, and green for SD9-BEC_12_ (Figure 1B). The entire fluorescence spectrum with excitation at 254 nm is shown in Figure 1 for all three sequences C_12_, SD9-OEC_12_, and SD9-BEC_12_ (Figure 1C–E, respectively).

The spectra have many similar fluorescent peaks. For example, the broad peak in the 300–400 nm part of the spectrum, which is assigned to the fluorescence of DNA bases [32]. The colors of AgNC observed in the trans-illuminator suggested that fluorescence peaks in the visible should be detected. SD9-OEC_12_ and SD9-BEC_12_ samples have a broad fluorescence in the visible region with two major peaks “red” at λ_R_ = 640 nm and “green”, λ_G_ = 525 nm for SD9-OEC_12_, and λ_G_ = 510 nm for SD9-BEC_12_, confirming that DNA template sequence has a direct effect on the fluorescent properties of silver nanoclusters. Closer inspection of the fluorescence spectra also revealed two shoulders to the major peaks at λ = 715 nm (red shoulder) and λ = 445 nm (green shoulder). Similar peaks are observed for C_12_–templated AgNC but the red fluorescence at 640 nm dominates the spectrum (Figure 1C). Table 1 shows the relative intensity of “green” to “red” peaks measured as areas of Gaussian fits for the two peaks in the spectrum plotted as fluorescence intensity vs. energy (Supplementary Figure S1). There is a clear trend of dominant red fluorescence in the following order: C_12_ > SD9-OEC_12_ > SD9-BEC_12_. The apparent color observed under UV excitation in the trans-illuminator thus represents a mix of two primary fluorescent peaks with different proportions—rather than a single color.

Despite the similarities of the templating sequences, C_12_, for all three designs, we observe large differences in the optical properties of the synthesized AgNCs. These differences go beyond the expected double intensity for SD9-BEC_12_ compared to SD9-OEC_12_. The intensity of “red” emission is similar, while “green” emission is dramatically enhanced for SD9-BEC_12_-templated AgNCs compared to SD9-OEC_12_. The SD9-OEC_12_ and C_12_ also exhibit noticeable differences. The “green” AgNCs have low fluorescence yield when C_12_ template is used. The “green” emission, however, seems to be largely present in SD9-OEC_12_ and even more intense in SD9-BEC_12_. It is tempting to speculate that the formation of “green” emitters is triggered by the double-stranded core SD9. Alternatively, a potential self-assembly of individual strands (Supplementary Figure S2) of SD9-OEC_12_ and SD9-BEC_12_, as predicted by NUPACK [33], may result in reorganization of the C_12_ strands in 3D space, thus changing the AgNC formation.

We have further characterized the properties of AgNCs templated with SD9-BEC_12_, SD9-OEC_12_, and C_12_ by employing fluorescence excitation-emission matrix (EEM) spectroscopy. The excitation/emission relationship of the optical response of the AgNCs can be a little complicated and a better way to represent such responses is through measuring the entire 3D excitation/emission matrix presented as contour maps. EEM has the advantage of showing the relationship between excitation, emission, and their intensities in the wide range of wavelengths, allowing for complete and quantitative characterization of sample’s fluorescence profile [25].

**Figure 2:**
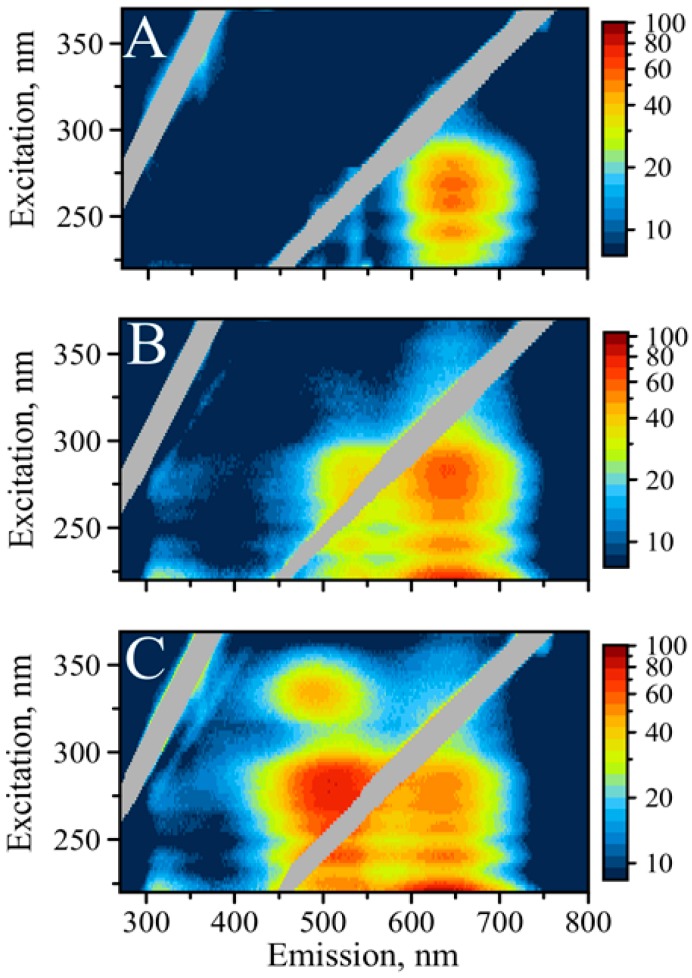
Excitation/emission maps of silver nanoclusters with excitation in UV: (**A**) AgNC templated on C_12_, (**B**) AgNC templated on SD9-OEC_12_, (**C**) AgNC templated on SD9-BEC_12_.

Figure 2 shows EEM maps for all three DNA sequences in the range 220–370 nm for the excitation while the entire emission spectrum in the UV and visible spanning 270–800 nm wavelengths was recorded. Some similarities in the behavior of emissions are obvious. For example, emissions of nucleobases spanning ~300 to ~350 nm was excited with UV: 220 to ~230 nm [32]. However, there are major differences observed in the emission of AgNCs in the visible spectral range excited via UV. Similar to the spectra shown in Figure 1, the red emission dominates C_12_, while green emission is dominant in the case of SD9-BEC_12_ template.

Close inspection of the emission peaks reveals that C_12_ has the maximum fluorescence at 645 nm, SD9-OEC_12_ at 640 nm, and SD9-BEC_12_ at 635 nm. Interestingly, this trend in the shift to lower wavelength also follows the increase in intensity of green fluorescence in the following order: C_12_, SD9-OEC_12_, and SD9-BEC_12_. These observations suggest that the “red” emitters synthesized using the three templates are either different species or the same species experiencing distinct surroundings.

While the “green” emission is practically absent in C_12_, it becomes detectable for SD9-OEC_12_ and is very intense for SD9-BEC_12_. The maximum of green emission for SD9-OEC_12_ is observed at 525 nm while for SD9-BEC_12_ it is observed at 510 nm, a noticeable 15 nm shift. Again, SD9- BEC_12_ template causes the fluorescence maximum to shift to lower wavelengths. Another major difference is an extra excitation band at 335 nm that only appears for the SD9-BEC_12_-templated AgNC and only for the green emission. This fluorescence peak has a maximum at 490 nm (“blue”). This serves as additional evidence of different surroundings of the emitting AgNC. We hypothesize that such differences are related to conformational heterogeneity of the DNA template induced by the presence of double-stranded SD9 core. Our EEM spectroscopic measurements reveal that the color of emission changes dramatically from template to template suggesting that AgNC of the same kind, “red” or “green”, experience different surroundings for the three templates used here.

It appears that the emission of AgNCs in the visible part of the spectrum is universally excited via DNA bases, which agrees well with published data [23]. It has been shown before that visible emission of AgNCs can be excited by both direct excitation into the visible band and by UV light into the absorption peak of DNA nucleobases [30,34]. A detailed study of UV excitation of DNA-templated silver nanoclusters suggests optical interactions between the DNA template and AgNC [23]. Excitation of AgNC fluorescence in the area of nucleobase absorption is a valuable feature of a DNA template. As such, optical properties of nucleobases can be beneficial for the excitation of AgNCs. The proximity of AgNC to DNA nucleobases, which not only nucleate and stabilize AgNC but also transfer the energy of the excitation, prompted more detailed studies of the phenomenon [23]. It has been proposed that DNA serves as an antenna in funneling the energy of UV light to fluorescence of AgNC [35], but the mechanism of energy transfer from the UV-excited nucleobases remains unclear. Nevertheless, the presence of coordinated DNA bases is necessary for the excitation of AgNC fluorescence by the UV light. Treating DNA-templated AgNC with DNAse reduces fluorescence intensity with time of treatment (Supplementary Figure S3). These results indicate an intimate relationship between optical properties of AgNC and templating DNA bases. In addition, during treatment “green” color disappears while “red” color still persists, although faintly, suggesting tighter connection of “green” emitters with DNA bases.

### 3.3. Optical Properties of AgNC under Visible Light Excitation

**Figure 3:**
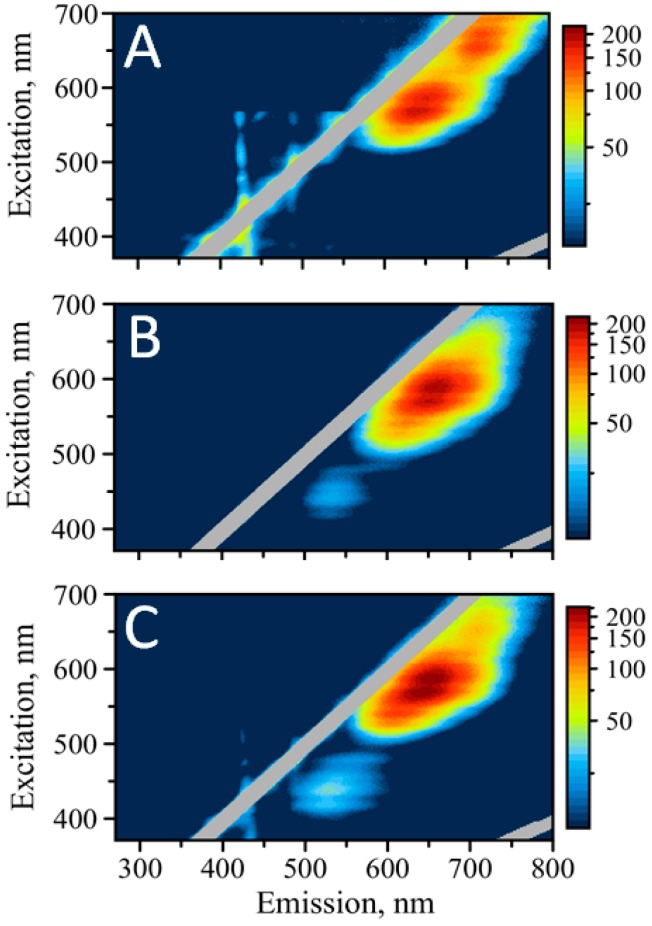
Excitation/emission maps of silver nanoclusters with excitation in the visible: (**A**) AgNC templated on C_12_, (**B**) AgNC templated on SD9-OEC_12_, (**C**) AgNC templated on SD9-BEC_12_.

Next, we measured excitation/emission maps for all three sequences while exciting in the range of visible wavelengths between 370 nm and 700 nm. While EEM with UV excitation reveal properties of the AgNCs that are in close proximity to nucleobases where efficient energy transfer from DNA bases is possible, the visible excitation probes optical properties of the nanoclusters in general, regardless of their association with the bases. Figure 3 shows the maps for C_12_, SD9-OEC_12_, and SD9-BEC_12_ (A, B, and C respectively).

It is obvious that there is one peak, which is similar for all three templates—the red emission above 600 nm. Interestingly, this peak appears as a duplet with maxima at 640 nm and 650 nm for all three samples. Another similarity is the emission at ~715 nm. It is well pronounced in C_12_ template but faintly present in both SD9-OEC_12_ and SD9-BEC_12_.

Consistent with the UV excitation, EEM maps for the visible excitation do not show any green fluorescence for the C_12_ template, while it is present for SD9-OEC_12_ and SD9-BEC_12_. We infer that such differences might be associated with the influence of the core SD9, which either stabilizes green emissive species or alters the conformation of C_12_–stretch in such a way that also allows for the formation of the clusters emitting in green.

Unlike other studies, we find that green and red emissions are not equivalent for the excitation in UV and the visible spectral region. Despite its bright appearance when excited in UV, green emission seems to be relatively silent when excitation is performed in the visible range of wavelengths. The maximum of emission is also shifted to lower wavelengths when comparing visible vs. UV excitation. The maximum of emission shifts by 10 nm for SD9-OEC_12_, with λ = 535 nm for visible excitation and λ = 525 nm for the excitation in UV. The maximum of emission for SD9-BEC_12_ shifts by more than 20 nm, with λ = 530 nm (for visible excitation) and λ = 510 nm (for UV excitation).

Additionally, red emission has very similar intensity for all three templates, while green clearly dominates the emission for SD9-BEC_12_-templated AgNCs when excited via UV. We hypothesize that SD9-OEC_12_ and SD9-BEC_12_ templates are capable of producing both types of AgNCs, green emitting and red emitting species with population largely shifted towards red species. The coordination with DNA bases in the case of green emitting clusters allows for more efficient energy transfer, larger quantum yield, and, therefore, greater fluorescence intensity of the green emitters when excited via DNA bases in UV.

One interesting feature of the red emission behavior for the AgNCs is that the emission maximum progressively shifts to the red as the excitation wavelength is increased starting from 515 nm all the way to ~700 nm (see Figure 3 and Figure S5). The magnitude of Stokes shift (λ_EM_ − λ_EX_), however, remains similar at ~70 nm throughout the visible part of the map. This bathochromic shifting in the emission band with the increase of the excitation wavelength resembles the phenomenon observed previously for polar organic fluorophores in “rigid” solutions [36,37,38]. The phenomenon of fluorescence dependence on the excitation wavelength is known as “red-edge excitation shift” (REES) [39]. This kind of dependence seems to be inconsistent with the Kasha’s rule, which states that the fluorescence spectrum should originate from the lowest vibrational level of the first excited singlet state irrespective of the excitation wavelength. Our AgNCs do not seem to obey this rule, exhibiting an obvious shift of emission to the red when excitation wavelength is increased, which is consistent with the REES effect. One possible explanation of such an effect in AgNCs is the “rigid” nature of clusters and their surroundings. The randomization of the local environment is not fast enough to allow for efficient relaxation of the excited state of the cluster. They are forced to emit not from the lowest level of the first excited singlet state but from the higher levels.

It is quite peculiar that no bathochromic shift is observed with the emission bands when excited via DNA bases. Quite smooth spectra with the same maxima are observed regardless of the change in excitation wavelength throughout the UV region. This observation suggests that Kasha’s rule is obeyed for the excitation of nanoclusters via UV, providing additional ground for speculation that the two populations of clusters are different in their photophysical properties, which stem from their different surroundings. The differences in optical properties between nanoclusters excited in UV and visible spectral range suggests a large degree of AgNC heterogeneity.

### 3.4. AFM Topography of DNA-Templated AgNC

**Figure 4:**
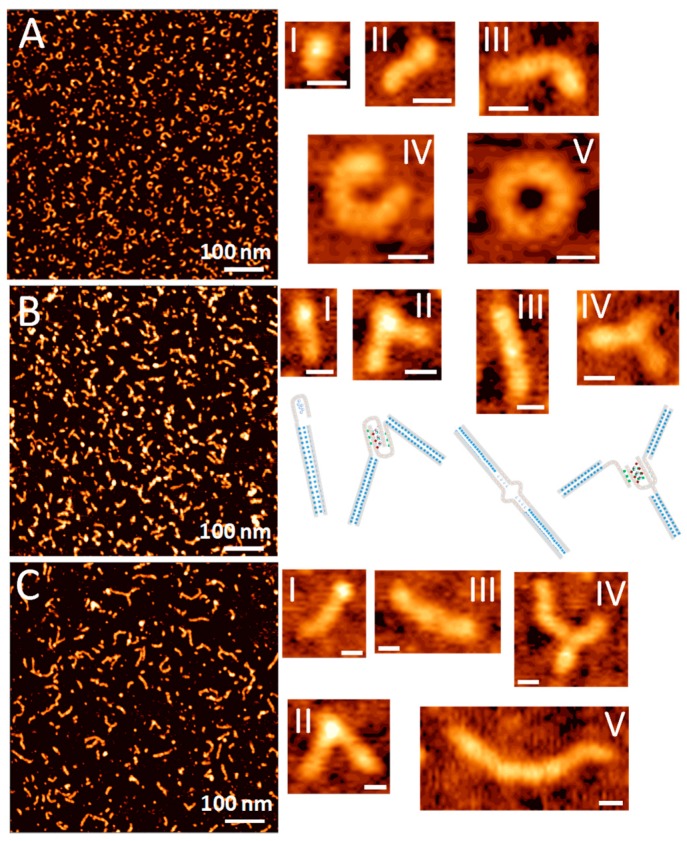
Atomic force microscopy (AFM) topography images of AgNC template with (**A**) C_12_, (**B**) SD9-OEC_12_, (**C**) SD9-BEC_12_. Insets show representative structures of the template sequences. Scale bar in all insets is 10 nm.

We have further investigated the morphology of the DNA-templated AgNC complexes using Atomic Force Microscopy. Figure 4 shows AFM topography images of samples for all three AgNC templating sequences: C_12_, SD9-OEC_12_, and SD9-BEC_12_. The AFM images of SD9-OEC_12_ show that templated silver nanoclusters adopt mostly small elongated shapes (Figure 4B, I) when deposited on mica substrate. The spherical structures were expected based on previously published data for long C-rich strands [40]. We assign these elongated shapes to a single SD9-OEC_12_. In addition to small elongated shapes, the SD9-OEC_12_ forms longer strands (Figure 4B, II), some of which have an angled junction. The total length of the entire SD9-OEC_12_ template is expected to be ~14 nm, if one assumes 0.34 nm base–base distance as in dsDNA. The measured contour length for elongated shapes in the AFM topography image exceeds the expected 14 nm. The strands with the angled junctions morphologies most likely represent adducts of two SD9-OEC_12_ templates joined together. Inset II in Figure 4B shows the length of the arms for angled shape is ~11 nm, which corresponds well to the expected length of 30 bp of the SD9 core. Inset III in Figure 4B shows another representative shape where two molecules form an adduct with a straight junction. This suggests that SD9-OEC_12_ sequences form a variety of shapes in solution as well as adducts between two or more molecules. Some shapes are tri-point or Y shapes with three strands joined together. Figure 4B inset IV shows one such representative shape, each strand measures ~14 nm indicating that the tri-point is a result of three connected SD9-OEC_12_ monomers.

The structures of SD9-BEC_12_-templated AgNCs are markedly different. There are still some elongated shapes observed in the AFM topography image shown in Figure 4C, but there are more longer strands in the image. The length of the strands for the SD9-BEC_12_ sample is rather heterogeneous with contour lengths up to 80 nm (Figure 4C, inset V). With extended C_12_ stretches, a single SD9-BEC_12_ molecule should measure ~18 nm assuming 0.34 nm base–base distance. The 80 nm long shape should be comprised of ~5 monomeric SD9-BEC_12_ molecules. Angled and straight dimeric/multimeric strands can also be found in topography images (insets II–IV in Figure 4B).

Longer assemblies are observed in the images of the C_12_ sample as well. Figure 4A shows an AFM image of C_12_-templated AgNCs. Elongated curved structures absolutely dominate the image. The length of the observed shapes exceeds the length of 12 base long oligonucleotide suggesting that the adducts of multiple C_12_ are formed. We further examined the cross-sections of the elongated shapes in the images. Typical AFM images in tapping mode produce the height of double-stranded DNA at around 0.5–0.8 nm [41,42,43]. While height values for most of the strands measured in our images do fall within this range, some areas show height values that exceed the height of the regular DNA duplex. A cross section in Figure 4B (inset II) for SD9-OEC_12_ indicates h = 0.7 nm in the arms of the dimeric adduct and h = 1.1 nm at the junction. Even visually, these two regions appear very different. The height of the point where two molecules are joined together suggests that the morphology of the DNA structure different from duplex is formed at the junction.

The observed differences in elongated shapes for C_12_, SD9-OEC_12_, and SD9-BEC_12_ are obviously due to the differences in the sequence that allow such distinct shapes to appear. We propose that the elongated strands observed are due to single-stranded (ss) C_12_ stretch forming a non-canonically bonded double-stranded DNA form via Ag+ linkage. Such a possibility has been demonstrated recently [44]. Alternatively, a tetra-stranded structure termed i-motif [45] could also form with the help of silver cations [46]. While connected via Ag^+^ linkage, double-stranded form is expected to appear as regular dsDNA in the AFM images. The i-motif, on the other hand, is composed of four strands and should appear higher on the image, as we observe at the junction (Figure 4B, inset II).

Both alternative structures differ from ss-DNA and yet serve as AgNC templates in a distinct way. While AFM provides unambiguous evidence for the formation of different shapes, we also observe remarkable differences in fluorescence between nanoclusters templated by C_12_, SD9-OEC_12_, and SD9-BEC_12_. We hypothesize that the differences in optical properties of silver nanoclusters are likely associated with the extent of non-canonical structure formations such as Ag^+^-C_12_ duplex or i-motif. Visually, C_12_ templates appear more curved, providing ground for speculation that curved geometry of template is responsible for the red emission of AgNCs in C_12_ while green emitters are predominately formed in linearly shaped templates (SD9-BEC_12_). The results of this section are an additional manifestation of how sensitive the optical properties are towards surroundings, namely the sequence of templating DNA and its secondary structure.

### 3.5. The First Step Towards Assembly

**Figure 5:**
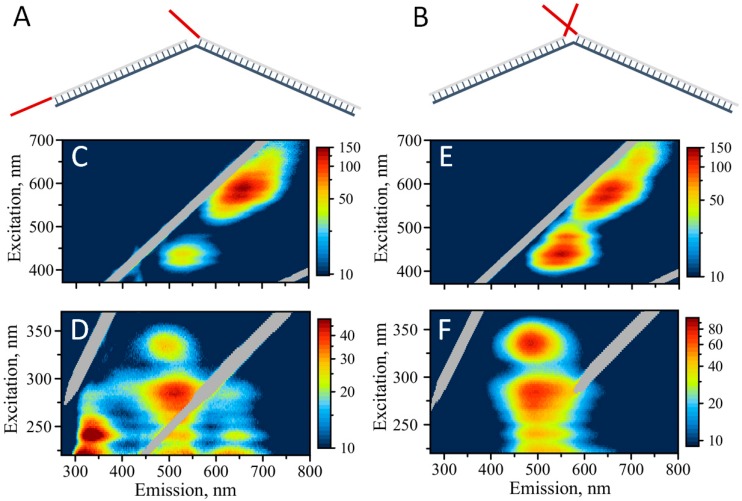
(**A**) Schematic representation of template design for the “forward” assembly, (**B**) schematic representation of template design for the “reverse” assembly. Templating C_12_ sequences are shown in red. Excitation/emission maps of silver nanoclusters: (**C**) AgNC templated on “forward” assembly with excitation in the visible spectral range, (**D**) AgNC templated on “forward” assembly with excitation in the UV, (**E**) AgNC templated on “reverse” assembly with excitation in the visible spectral range, (**F**) AgNC templated on “reverse” assembly with excitation in the UV.

Dimeric assembly with a dual cluster templating sequence can represent a first step towards a larger DNA-based network. Figure 5 schematically illustrates such a dimeric design. We explored two different variants of the dimeric assembly: (1) “Forward” design, where C_12_ templating stretches are separated by 30 bp double-stranded core sequences—SD9 (Figure 5A), and (2) “reverse” design, with C_12_-templating sequences brought together by this assembly (Figure 5B). Fluorescence properties of AgNCs templated by the “forward” assembly resemble well the properties of AgNCs formed with SD9-BEC_12_ sequence including the unusual 335/490 nm “blue” peak (Figure 5C,D). The intensities of all the peaks, both in UV-excited emission and visibly-excited emission for the “forward” assembly are exactly the same as for SD9-BEC_12_, confirming the similarity of the structure and conformation of DNA and AgNCs.

It is quite interesting that the optical properties of the resultant assemblies do not resemble SD9-OEC_12_ but appear similar to SD9-BEC_12_ including the prevalence of green fluorescence and the “blue”—335/490 nm peak. Visible excitation of emission produces a similar map as for SD9-BEC_12_ and SD9-OEC_12_ with the characteristic double peak: 570/650 nm and 590/660 nm. In addition, a small peak at 435/530 nm is observed, similar to both SD9-OEC_12_ and SD9-BEC_12_ templates.

Dramatic changes of the AgNC optical properties are observed when “reverse” design was utilized, Figure 5E,F. The enhancement of the green emission is quite obvious. There are only slight remnants of the red fluorescence while green emission dominates the 2D map when excited in the UV (Figure 5F). The 335/490 nm “blue” peak is now dominant in the excitation/emission map, providing additional clues to the origin of this peak, which are described in the Discussion section. The 2D excitation/emission map with visible excitation for the “reverse” design appears to be different from the one for “forward” design. “Green” peak is intensified and shifted to longer wavelengths in the “reverse” assembly. In addition, “red” fluorescence at ~700 nm is enhanced in the “reverse” design compare to “forward” assembly. Similar to individual templates, the maxima of emission in the assemblies do not coincide when AgNCs are excited via UV and visible, as clearly observed in the maps. “Red” emission is intensified with visible excitation and “green” is shifted to lower wavelengths when excited via UV.

## 4. Discussion

NA-based tools of nanotechnology open large prospects in designing and controlling the size and shape of nanostructures. DNA can serve as a template for the controlled synthesis of novel nanomaterials that are arranged in a highly organized network of functional entities, exemplified in this work by AgNCs. Beneficial novel properties may result from placing AgNCs into nanostructures with organized patterns. Examples of such beneficial properties are the use of AgNCs as logic optical gates [47], optoelectronics [15], bioimaging, or detection of specific nucleic acid sequences where the signal is amplified due to optical coherence of the signal from AgNCs organized in patterned nanostructures. Additionally, controlled AgNC formation can be used as the means of label-free visualization of NA supra-assemblies and nanomaterials.

One of the questions that we address with this study is, what would be the properties of AgNCs when they are assembled into larger networks using DNA as an architectural element? The effects needed to be probed are the close proximity of the DNA on the optical properties of templated AgNCs such as double-stranded core, loose ends of nucleic acids, and other structural elements used in the field of DNA nanotechnology [48]. Our choice of template sequence was single-stranded cytosine stretch—C_12_, which has been previously shown to produce silver nanoclusters with high yield of fluorescence in the visible range of wavelengths [30]. Additional double-stranded region was placed next to C_12_ mimicking the nanostructure design when larger assembly is created. Double-stranded DNA show no template activity unless modified to contain a mismatched site, an abasic site, a gap, a bulge, or a loop [13]. Silver nanoclusters generated by the double-stranded templates exhibit negligible fluorescence signal. We verified the inability of SD9 core to generate and stabilize any type of clusters by running a control experiment where SD9 core duplex was mixed with AgNC forming components: Ag^+^ and NaBH_4_. No detectable fluorescence was observed without the C_12_-templating sequence (Supplementary Figure S4).

While C-rich sequences have high affinity to silver, which makes them very good templating sequences for AgNC synthesis, they also have the ability to form alternative non-canonical DNA structures. For example, a duplex stabilized by silver in a C-Ag-C pairing rather than canonical Watson-Crick [49]. Such structures are quite stable, Ag^+^ bridges the N3 atoms of the C bases resulting in the C-Ag-C pairing link [50]. Another example of non-canonical form is the i-motif where semiprotonated C-rich oligonucleotides are arranged in a tetra-stranded structure [51]. Typically, the formation of i-motif structures is favored at mild acidic conditions due to protonation of N3 in the cytosine. The entire structure is stabilized via hydrogen bonds between C^+^–C in the semi-protonated base pairs. However, silver cation can join C strands in a C-Ag^+^-C bridge even at neutral pH values [46]. The affinity to silver is so specific that an Ag^+^ detection has been proposed based on i-motif formation due to the presence of Ag [52].

At neutral pH, the formation of alternative structures leading to elongated shapes observed in AFM experiments depends critically on the silver content in the solution. Our data suggest that the elongated strands are linked together via a non-canonical structure with the help of Ag^+^. We considered both versions of the possible structures: The silver-stabilized duplex between two C_12_ stretches and the i-motif. The duplex between C_12_ strands stabilized by silver is not expected to appear much different from regular DNA duplex in the AFM topography images. An example is round shaped adducts observed in AFM topography images for the C_12_ sequence. While it is clearly an adduct of many molecules of C_12_, the height of the structure appears similar to dsDNA at ~0.7 nm. Contour length analysis of the shape shown in Figure 4A, inset V (L_C_ = 30 nm) suggests that up to 20 molecules of C_12_ need to join together to form such a shape. On the other hand, the i-motif is structurally different from single-stranded or double-stranded DNA. The four-stranded nature of the i-motif increases the lateral size of the structure, which can be easily identified using AFM. Indeed, AFM allowed us to recognize the features that are larger than DNA duplex. Examples of structures formed with i-motif are dimeric and trimeric adducts with junctions that exceed sizes of ds-DNA (Figure 4B, insets I and II). Figure 4 presents our vision as to how such i-motif junctions can form. It has been shown that both inter-molecular packing and intra-molecular folding of strands into i-motif is possible [53]. Several studies have already started exploring the use of i-motif as a structural element in DNA nanotechnology [11].

The i-motif formation results in a more compact shape than duplex. It has a shorter distance between the bases, 0.31 nm as compared to 0.34 nm in a regular double strand [40,54]. Therefore, i-motif creates a denser population of silver cations holding the i-motif together. The alignment of silver cations and the shape of the resultant nanoclusters templated by the i-motif are expected to be different from those of both single-stranded C_12_ and double-stranded C_12_-Ag-C_12_. Because of such difference, the i-motif structure should also affect optical properties of templated AgNCs. It has been stated previously that i-motif structures primarily produce red emissive AgNCs [55]. Further studies using single nanocluster fluorescence measurements and their correlation with AFM imaging will unambiguously assign the color and photophysics of AgNCs to the template type, specifically i-motif, and we are currently considering these single nanocluster studies.

The measured excitation/emission matrices for C_12_, SD9-OEC_12_, and SD9-BEC_12_ sequences indicate that there are mostly two distinct color clusters—“green” and “red”. Previously, the distinct fluorescence peaks have been associated with clusters of different sizes [30]. DNA templates are capable of stabilizing various (Ag)_N_ clusters where N is the number of silver atoms forming a nanocluster. It is commonly accepted that the DNA templated (Ag)_N_ nanoclusters are rod like in shape and include both neutral Ag^0^ atoms and charged Ag^+^ [56]. Recent study revealed that a “magic number” of neutral silver atoms is required for distinct “green” and “red” fluorescence to occur. Namely, it has been inferred that four neutral atoms produce green fluorescence and six neutral Ag atoms produce red fluorescence regardless of the number of Ag^+^ [57]. In general, larger AgNCs emit at longer wavelengths [58]. Therefore, it seems appropriate in our case to assign green fluorescence to AgNCs of smaller cluster size with four Ag^0^ and red fluorescence at 640 nm to larger clusters with six Ag^0^.

The environment of the clusters composed of nucleobases, although very important, is poorly understood. The tuning of fluorescent properties of AgNCs is often done only empirically. Recent studies have tried to look into a deeper understanding of the role different nucleobases play in optical/photophysical properties of AgNCs [23,59]. When placing AgNC into larger assemblies of nucleic acid structures, several factors may play a role in altering the AgNC fluorescence: (1) Templating sequence, (2) adjacent double-stranded sequence, and (3) the overhang sequence. As all three are contributing factors to fluorescence, changing them provides additional degrees of freedom for tuning the fluorescence properties of AgNCs. A vivid example of the effect of non-templating sequence on optical properties of AgNCs is the presence of overhang strands. G-rich overhangs produce drastic changes in excitation, emission, and quantum yield of fluorescence [18]. Our results demonstrate that simply changing the direction of dimeric assembly from “forward” to “reverse” drastically increases “green” fluorescence when excited via DNA bases with UV. “Red” emitting species are still formed in the “reverse” assembly, as indicated by the excitation in the visible, but the UV excitation channel via DNA bases is completely turned off for the “red” emitting nanoclusters. It has been established that bases are electronically coupled to silver nanoclusters. One manifestation of such coupling is the AgNC emission when excited at nucleobases’ UV absorption. The heteroatoms of bases, particularly N7, N3, and N2 change the electronic environment of the cluster. This, in turn, stabilizes particular emissive levels, enhances the fluorescence quantum yield, and increases fluorescence lifetime by several nanoseconds [59]. Our observations point to the complexity of the electronic structure of the nanoclusters and the abundance of possibilities of the de-excitation processes (Supplementary Figure S5).

We hypothesize that the “green” emitting nanoclusters are tightly associated with DNA bases leading to their selective UV excitation. This is contrary to the “red” emitting clusters where association is not as tight, resulting in preferential excitation in the visible. More evidence of tight DNA association with “green” emitting clusters is the apparent lack of red-edge emission shift (REES) for these AgNCs excited in the visible. In general, REES is observed when the process of internal conversion is very slow compared to fluorescence lifetime. REES has been observed for polar fluorophores in viscous media [38]. Relaxation due to solvent molecule reorientation is not fast enough within the fluorescence lifetime, leading to the observation of the emission maximum shifting to the red. No REES is observed for the AgNCs, both “green” and “red” emission, when excited via UV. This indicates that nanoclusters tightly associated with DNA bases experience an environment that provides effective randomization. The “red” emission excited in the visible has a clear REES effect. On the other hand, the maximum of the 550 nm peak in the fluorescence of AgNCs excited in the visible for the “reverse” design appears to be shifting only slightly to the red but not as fast as the emission in the ~600–700 nm region. This situation appears somewhat intermediate between the “rigid” and “flexible” environments, providing some degree of randomization of the surroundings. We hypothesize that this effective randomization originates from tighter association of “green” nanoclusters with DNA bases. While the observed differences between “green” and “red” emitting species indicate a distinct AgNC environment, additional time-resolved detailed spectroscopic measurements will help to more fully describe this phenomenon. One of the most intriguing results of our study is the appearance of the “blue” emitting species, 335/490 nm. These species are excited via UV but emit in the visible spectral range. The magnitude of Stokes shift for these species, ∆λ ≈ 155 nm, is almost double that for nanoclusters excited in the visible range with ∆λ ≈ 70 nm, but not nearly as high as the Stokes shift observed for UV excited emission, ∆λ up to 300 nm for “green” or ∆λ up to 420 nm for “red”, indicating uniqueness of these hybrid species. The formation of the “blue” emitters is favored whenever C_12_ templating sequence is placed at the 5′ end of the double-stranded core. This is the case with SD9-BEC_12_ as well as both dimer assemblies. We have verified the link between 5′ template and blue nanocluster formation by placing C_12_ at 5′ in the SD9-5′-OEC_12_. There is a clear peak in the blue indicating the formation of the “blue” emitters (Supplementary Figure S6). We, therefore, conclude that C_12_ sequence placed at 5′ somehow stimulates the formation of the “blue” emitters, perhaps by adopting favorable conformation.

Overall, our results suggest a high degree of heterogeneity of synthesized AgNCs. Since the resulting AgNCs are so heterogeneous, it might be very difficult to purify a single population of AgNCs, which will exhibit homogeneous optical properties as the environment variation affects individual clusters. Therefore, ensemble-based measurements may not be an appropriate choice of method for studying properties of AgNCs. It is reasonable to suggest that single nanocluster spectroscopic studies should provide a clearer understanding of the AgNC optical properties free of complications originating from the heterogeneous nature of nanoclusters in ensemble.

## 5. Conclusions

In summary, our studies offer a detailed characterization of AgNC synthesized using C_12_-based template sequences designed for construction of larger DNA-based assemblies. We have shown the possibility of combining silver nanoclusters into larger assemblies of nucleic acids using a simple design containing a single-stranded template and a double-stranded core. A full excitation/emission data matrix has proven to be an effective tool in exploring the complex optical behavior of silver nanoclusters templated on nucleic acid sequences. Our findings indicate that close proximity of double-stranded region changes preferential formation of “red emitters” with λ_EM_ = 640 nm observed on C_12_ sequences to the formation of “green emitters” with λ_EM_ = 510 nm when double-stranded region is present. Further detailed exploration of NA templates, specifically their conformation and the possibility of non-canonical structure formation, will facilitate our understanding and control of AgNC properties. Our results also show that the fluorescence of AgNCs can be modulated by controlling the position of the AgNCs within the dimeric assembly. Placing nanoclusters closer together dramatically enhances the fluorescence of AgNCs, but also changes the electronic structure of the nanoclusters, primarily affecting the excitation/emission of the “green” and the “blue” emitters. Our observations point to the complexity of the electronic structure of the nanoclusters and the abundance of possibilities of the de-excitation processes. This in turn allows for fine-tuning of fluorescent properties of AgNCs. Effective randomization of the local environment for the “green” emitting species, as manifested by the reduction in REES, is primarily observed for the clusters placed in proximity. We envision a construction of large assemblies using nucleic acids as architectural templates with unique tunable fluorescent properties of AgNCs that could be used in novel materials, devices, and diagnostic applications.

## Figures and Tables

**Table 1:** Relative ratios of fluorescence intensities for “green” and “red” emitters obtained from spectra recorded using 254 nm excitation. The numbers were obtained as areas under the Gaussian fits to intensity of fluorescence plotted vs. energy (shown in Supplementary Figure S1).

F_MAX, nm_	C_12_	SD9-OEC_12_	SD9-BEC_12_
510-525 (green)	13	42	69
640 (red)	87	58	31

## Supplementary Materials

The following are available online at https://www.mdpi.com/2079-4991/9/4/613/s1, Figure S1: Fluorescence spectra of AgNCs recorded with 254 nm excitation wavelength and plotted as fluorescence intensity vs. energy (eV), Figure S2: Predicted secondary structures using NUPACK, Figure S3: Fluorescence intensity change of C_12_-templated AgNCs treated with RQ1 DNase, Figure S4: Full excitation/emission map for double-stranded SD9-core template, Figure S5: Full excitation/emission energy map of AgNCs templated on SD9-OEC_12_ sequence with schematic Jablonski diagrams showing de-excitation pathways for the “red” and the “green” emitters, Figure S6: UV excitation/emission map for SD9-5′-OEC_12_ templated AgNCs.
