# Supplementary material for: First Step Towards Larger DNA-Based Assemblies of Fluorescent Silver Nanoclusters: Template Design and Detailed Characterization of Optical Properties
Source: Nanomaterials (Basel). 2019 Apr 13;9(4):613. doi: 10.3390/nano9040613 (PMC6523636; doi:10.3390/nano9040613)
Supplement: Supplementary file 1 [file nanomaterials-09-00613-s001.pdf]

Supplementary Materials

First Step Towards Larger DNA-Based Assemblies of Fluorescent Silver Nanoclusters: Template Design and Detailed Characterization of Optical Properties

Liam Yourston [1], Alexander Lushnikov [2], Oleg Shevchenko [3], Kirill Afonin [3] and Alexey V. Krasnoslobodtsev [1,2,*]

[1] Department of Physics, University of Nebraska Omaha, Omaha, NE 68182, USA; lyourston@unomaha.edu
[2] Nanoimaging Core Facility at the University of Nebraska Medical Center, Omaha, NE 68198, USA; alushnikov@unmc.edu
[3] Nanoscale Science Program, Department of Chemistry, University of North Carolina at Charlotte, Charlotte, NC 28223, USA; oshevche@uncc.edu (O.S.); kafonin@uncc.edu (K.A.)
* Correspondence: akrasnos@unomaha.edu or a.krasnoslobodtsev@unmc.edu; Tel.: +1-402-554-3723.

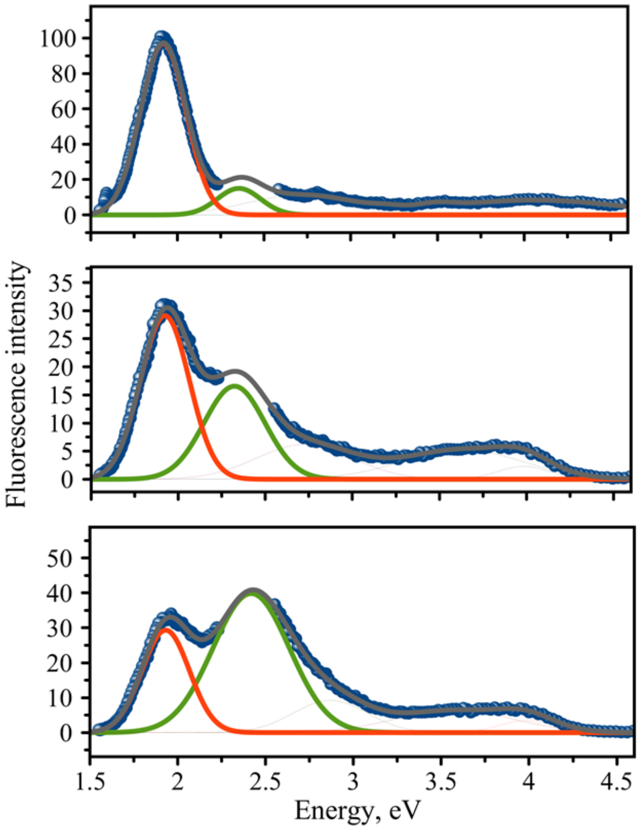

Figure 1. Fluorescence spectra of AgNCs recorded with 254 nm excitation wavelength and plotted as fluorescence intensity vs energy (eV) for the following templates from top to bottom: C_{12}, SD9-OEC_{12}, SD9-BEC_{12}.

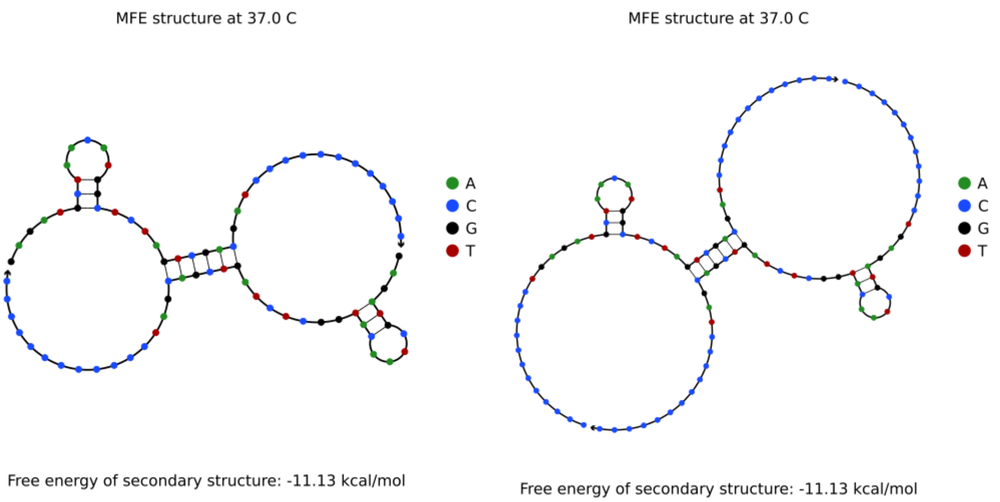

Figure 2. Predicted secondary structures of self-assembled SD9-OEC12 (left) and SD9-BEC12 (right).

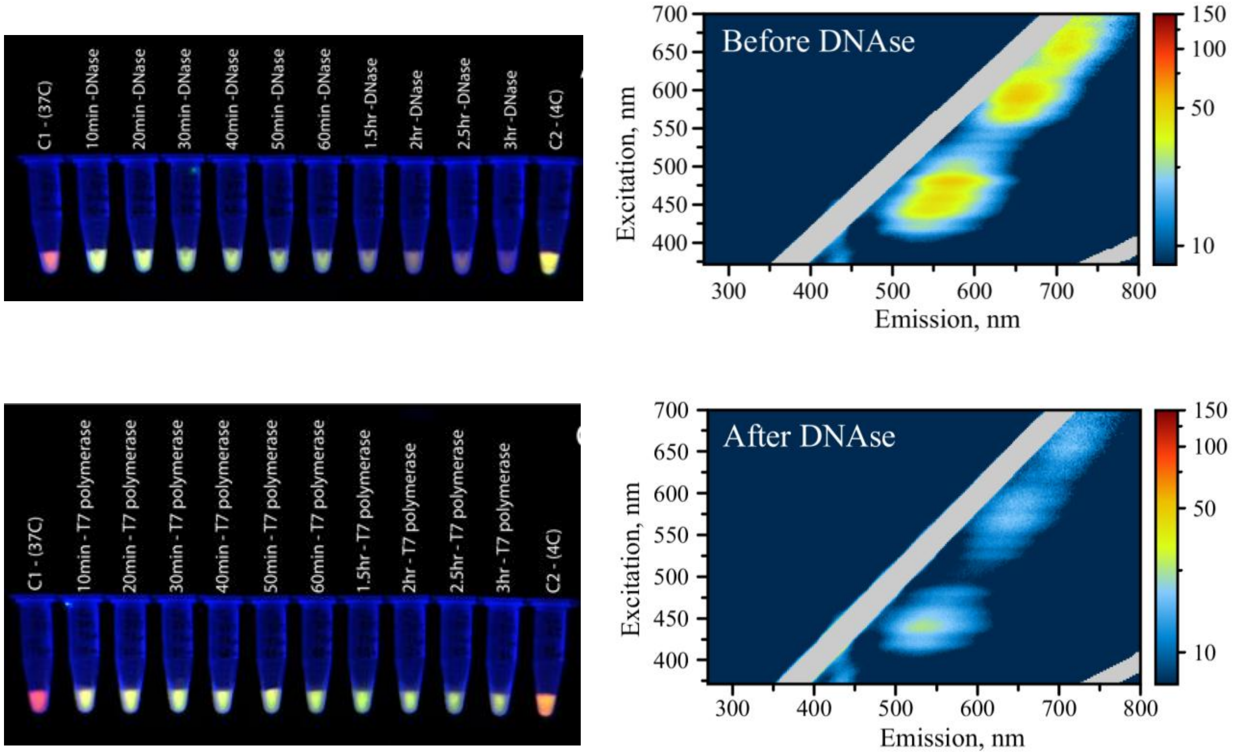

Figure S3. Fluorescence intensity of C12-templated AgNCs under UV illumination. Top panel shows time evolution of AgNC fluorescence in samples treated with RQ1 DNase. Top panel shows time evolution of AgNC fluorescence in samples treated with T7 RNA polymerase. Control samples, C1 and C2, in each of the panel were not treated with enzymes and were incubated either at 37 °C or at 4 °C in the dark for C1 and C2 respectively. Panel on the right shows full EEMs with excitation in the visible spectral range showing dramatic reduction in fluorescence intensity after treatment with DNAse.

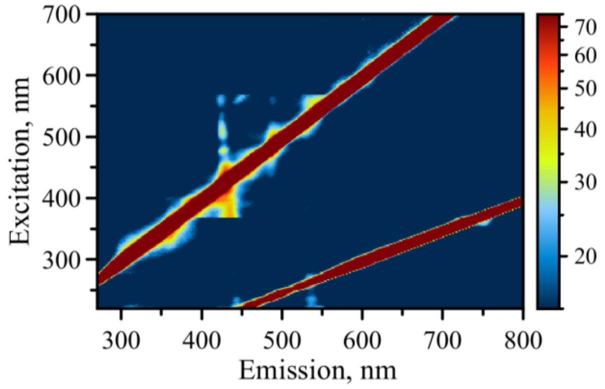

Figure S4. Full excitation/emission map for double-stranded SD9-core. At the same experimental conditions used in this study dsSD9-core does not template fluorescent AgNCs as evident from the intensity of fluorescence in both UV and visible spectral range.

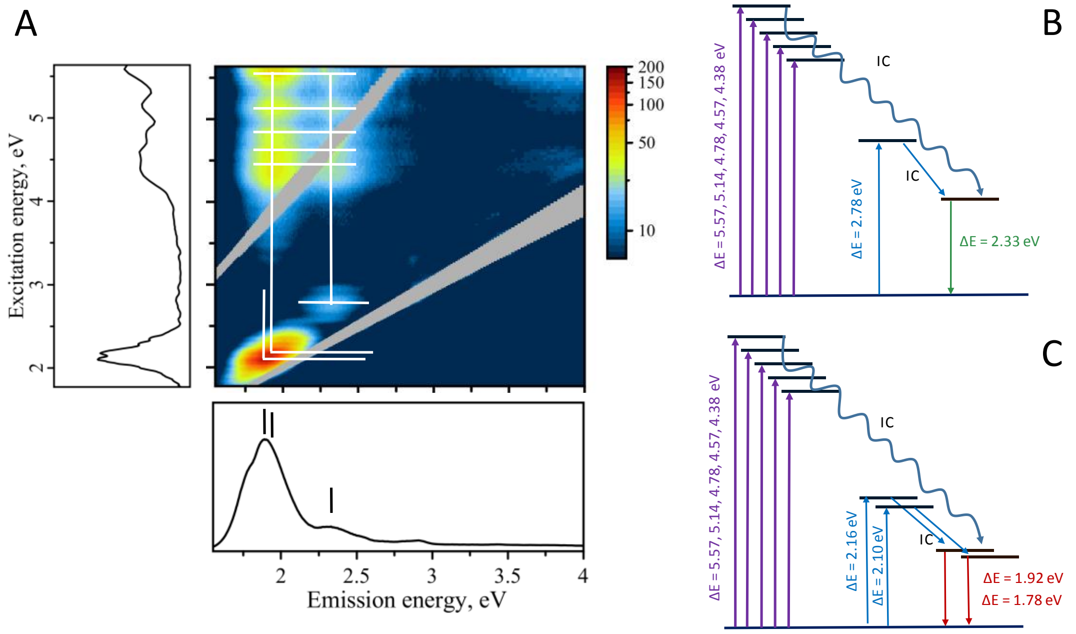

Figure S5. Full *excitation/emission energy* map of silver nanoclusters: (**A**) Excitation – emission matrix of AgNCs templated on SD9-OEC12 sequence, (**B**) Schematic Jablonski diagram for observed transitions in "green" emitting AgNCs on SD9-OEC12, (**C**) Schematic Jablonski diagram for observed transitions in "red" emitting AgNCs on SD9-OEC12.

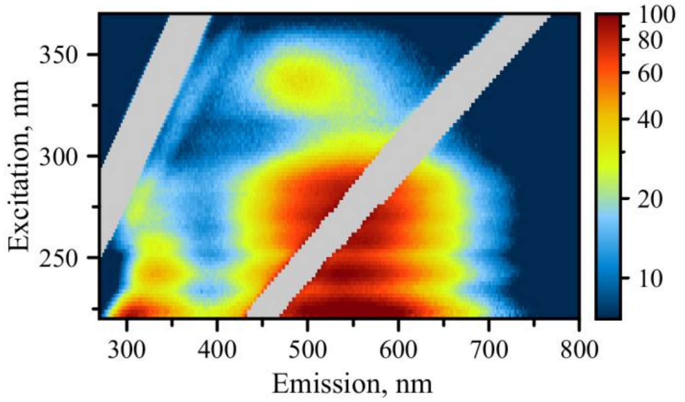

Figure S6. UV excitation/emission map for SD9-5′-OEC12 template. 5′ located templating sequence induces the formation of "blue" fluorescent AgNCs with 335 nm excitation maximum and 490 nm emission maximum.
